# Mechanical morphotype switching as an adaptive response in mycobacteria

**DOI:** 10.1126/sciadv.adh7957

**Published:** 2024-01-03

**Authors:** Haig Alexander Eskandarian, Yu-Xiang Chen, Chiara Toniolo, Juan M. Belardinelli, Zuzana Palcekova, Lesley Hom, Paul D. Ashby, Georg E. Fantner, Mary Jackson, John D. McKinney, Babak Javid

**Affiliations:** ^1^ Division of Experimental Medicine, University of California San Francisco, San Francisco, CA 94143, USA.; ^2^ Molecular Foundry, Lawrence Berkeley National Laboratory, Berkeley, CA 94720, USA.; ^3^ School of Life Sciences, Swiss Federal Institute of Technology in Lausanne (EPFL), 1015 Lausanne, Switzerland.; ^4^ Mycobacteria Research Laboratories, Department of Microbiology, Immunology and Pathology, Colorado State University, Fort Collins, CO 80523-1682, USA.; ^5^ Materials Science Division, Lawrence Berkeley National Laboratory, Berkeley, CA 94720, USA.; ^6^ School of Engineering, Swiss Federal Institute of Technology (EPFL), 1015 Lausanne, Switzerland.

## Abstract

Invading microbes face a myriad of cidal mechanisms of phagocytes that inflict physical damage to microbial structures. How intracellular bacterial pathogens adapt to these stresses is not fully understood. Here, we report the discovery of a virulence mechanism by which changes to the mechanical stiffness of the mycobacterial cell surface confer refraction to killing during infection. Long-term time-lapse atomic force microscopy was used to reveal a process of “mechanical morphotype switching” in mycobacteria exposed to host intracellular stress. A “soft” mechanical morphotype switch enhances tolerance to intracellular macrophage stress, including cathelicidin. Both pharmacologic treatment, with bedaquiline, and a genetic mutant lacking *uvrA* modified the basal mechanical state of mycobacteria into a soft mechanical morphotype, enhancing survival in macrophages. Our study proposes microbial cell mechanical adaptation as a critical axis for surviving host-mediated stressors.

## Teaser

Mycobacterial mechanical morphotype switching enhances survival during macrophage infection.

## INTRODUCTION

Cell surface elasticity outlines the physical bounds within which microbial life manifests (*1*, *2*). Bactericidal stresses drive the biophysical collapse of cell surface mechanical properties maintaining cell wall integrity, culminating in lysis (*2*–*4*). Preventing cell lysis is paramount to cell survival, particularly during host pathogenesis when microbes encounter diverse stresses inflicting physical damage. Maintaining a balance of cell mechanical forces is critical to survival. Here, we sought to define the fundamental principles of mycobacterial cell surface mechanical properties to reveal how mycobacteria physically tolerate the stresses of intracellular infection.

Atomic force microscopy (AFM) has previously been used to characterize perturbations of cell wall components at the bacterial cell surface (*2*, *3*, *5*, *6*). More recently, long-term time-lapse AFM (LTTL-AFM) of mycobacteria has defined a synergistic role for cell biomechanics and molecular components influencing fundamental proliferative cell processes, such as division site selection, cell cleavage, and pole elongation (*3*, *4*, *7*).

Here, using LTTL-AFM and differential buoyancy fractionation, we identify “soft” and “hard” mechanical morphotypes in mycobacteria representing cell states in which basal mean cell surface stiffness is shifted in *Mycobacterium smegmatis* (Msm) and *Mycobacterium abscessus* (Mab). Using a combination of genetic and pharmacological approaches, we identified that mycobacteria with soft mean cell surface stiffness increased survival within macrophages, suggesting a role for microbial mechanical cell states in adapting to host-mediated stressors.

## RESULTS

**Fig. 1.**
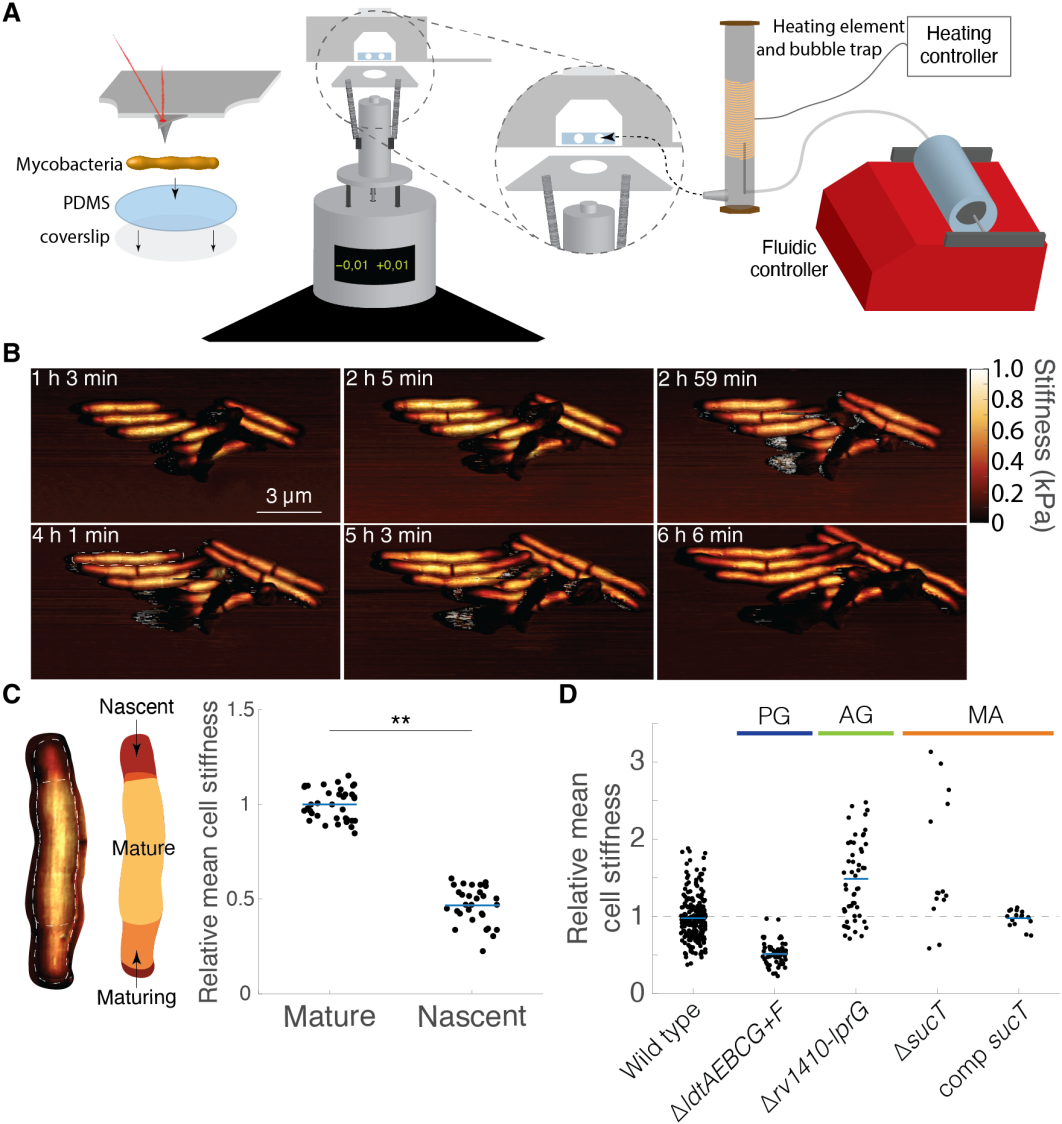
The mycobacterial cell wall controls surface mechanical maturation. (**A**) Schematic representation of the LTTL-AFM imaging system depicting the setup for mycobacterial sample scanning (left) and the integrated heating element and fluidic systems (right) for controlling the environmental conditions while imaging with a Bruker MultiMode 8 scanner. (**B**) Time series of three-dimensional AFM height images overlaid with mechanical stiffness (DMT modulus) images for WT Msm. (**C**) Relative cell surface stiffness of nascent cell wall material as compared to mature material (*n* = 30). (**D**) The relative mean cell surface stiffness of cell wall mutants in Msm—data adapted from (*1*, *12*, *13*). **P < 0.01 by unpaired *t* test.

We used LTTL-AFM to dynamically characterize the range in cell surface stiffness in Msm along the long axis of the cell (Fig. 1A). Growth in Msm consists of pole elongation and subcellular changes in cell surface stiffness, predominantly at subpolar regions (Fig. 1, A and B; movie S1; and fig. S1). Elongation results from an expansion of cell volume by addition of nascent cell wall material near the poles (*1*, *3*, *7*). The elongating mycobacterial cell surface consists of three discrete regions of mechanically distinct material (fig. S1). Most of the cell surface (~75%), emanating from mid-cell and excluding the poles, is composed of stably rigid cell surface material (Fig. 1C and figs. S1 and S2A). Previous studies suggest that l,d-transpeptidase–dependent cross-linking of peptidoglycan is critical for maintaining a stably rigid cell surface (*1*). Subpolar regions comprise ~25% of the cell length [~20% at the old pole and 7% at the new pole, pre-NETO (new end take-off)] and harbor the addition of nascent, non–cross-linked peptidoglycan (*1*, *8*) where surface material begins to gradually increase in stiffness (Fig. 1C and figs. S1 and S2A), revealing a process of “mechanical maturation.” The slow growth represented by pre-NETO pole elongation dynamics at the new pole restricts the subpolar region where mechanical maturation of cell surface material takes place. Pole elongation dynamics control the shift in mechanically rigid zones toward mid-cell (*7*).

These findings expand the description of mycobacterial growth based on pole elongation and mechanical maturation. Previous studies described mycobacterial growth to be tightly controlled spatially by the addition of nascent cell wall material at subpolar regions (*9*) and temporally (*7*) as a function of biphasic pole elongation dynamics. LTTL-AFM imaging has revealed the manifestation of a wave-form cell surface morphology culminating in wave troughs and peaks, which emerge as a result of pole elongation, and establish licensed sites for division site selection (*3*). Here, LTTL-AFM reveals a mechanically dynamic cell surface organized into zones of hardening soft material at polar and subpolar regions, corresponding to zones where new cell wall material is directed (*1*), and zones of stably ‘hardened” material near mid-cell.

Perturbation of the mycobacterial cell wall, composed of multiple layers that mechanically maintain cell morphology as load-bearing units (*10*, *11*), has opposing effects on the cell-surface mechanical state. Cells exhibiting defective peptidoglycan cross-linking due to deletion of l,d-transpeptidases (∆*ldtAEBCG*+*F*) results in decreased mean cell surface stiffness, culminating in a turgor pressure-driven gradual bulging near the new pole (Fig. 1D and movie S2) (*1*). By contrast, deleting genes involved in the maturation and export of arabinogalactan and lipoarabinomannan (∆*Rv1410*-*lprG* and ∆*sucT*) results in increased mean cell surface stiffness, likely by exposing the AFM cantilever to a more load-bearing unit of the cell wall (Fig. 1D) (*12*, *13*). Molecular perturbation of biosynthesis processes for discrete wall layers results in a more homogeneous cell surface, without obviously mechanically distinct zones of cell surface material, and shifts the mean cell surface stiffness to either soft or hard mechanical cell states.

To measure dynamic shifts in mean cell surface stiffness, we used a pharmacological approach. Mycobacteria treated with isoniazid (INH) gradually drove a twofold increase in mean cell surface stiffness and collapsed phenotypic heterogeneity (fig. S3A). Mechanically distinct zones of cell surface material were reduced by INH-induced reductive division and arrest of pole elongation (fig. S2B) while maintaining variance in cell surface stiffness (fig. S4A). Resumed pole elongation resulted in the addition of nascent material undergoing a gradual mechanical maturation returning the mean cell surface stiffness to untreated levels (figs. S3A and S4 and movie S3). Treatment with the bacteriostatic cyanide derivative, carbonyl cyanide *m*-chlorophenyl hydrazone (CCCP), caused a rapid ~35% decrease in mean cell surface stiffness (10 kPa min^−1^). Decreased stiffness was partially reversed by exchanging the tonicity of the growth medium with water (fig. S3B), suggesting that decreasing mean cell surface stiffness can be mediated by manipulating adenosine 5′-triphosphate (ATP)–dependent control of turgor pressure. CCCP treatment results in a reduction of the variance in cell surface stiffness (fig. S4B). Release from CCCP treatment resulted in the recovery of cell surface stiffness (~5 kPa min^−1^), before the resumption of elongation (figs. S3B and S5 and movie S4). Bedaquiline (BDQ), a clinically relevant antibiotic in the treatment of *Mycobacterium tuberculosis*, which inhibits ATP generation (similarly to CCCP), equally provoked a ~20% reduction in mean cell surface stiffness (fig. S3C). In contrast, mitomycin C, an antibiotic provoking DNA damage, does not cause a shift in mean cell surface stiffness (fig. S3D). The trajectory of mechanical shifts in mean cell surface stiffness is influenced by distinct physical cell properties. Perturbing the inner cell wall resulted in a decreased mean cell surface stiffness while perturbations to the outer cell wall and cytosolic tonicity drove bacteria into a hard mechanical cell state.

**Fig. 2.**
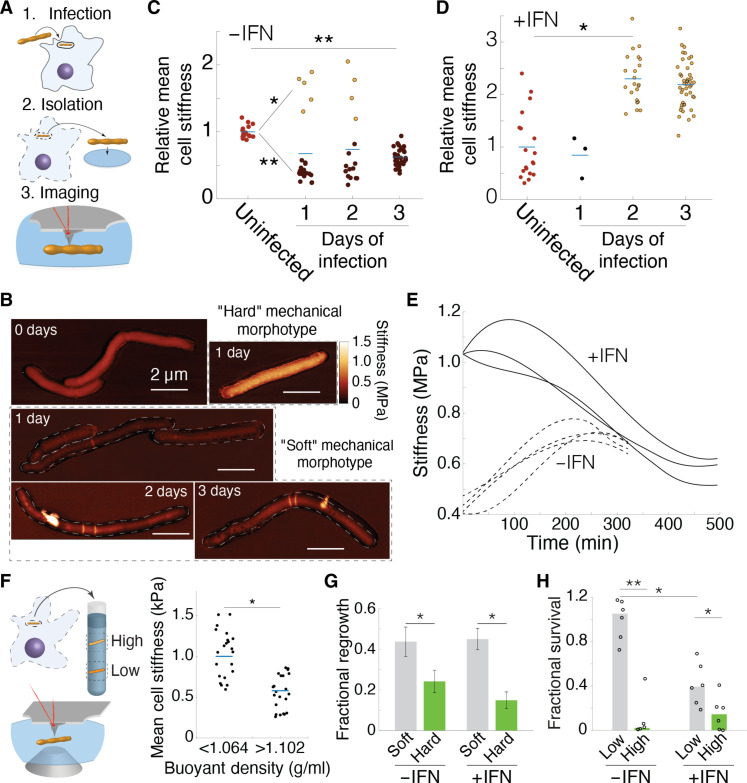
Host macrophage internalization drives selection of distinct mycobacterial mechanical morphotypes. (**A**) Schematic representation of three critical steps for quantifying surface mechanical properties of macrophage-intracellular mycobacterial cells. (**B**) Three-dimensional AFM height images overlaid with stiffness (DMT modulus) images of mycobacterial cells isolated from macrophages during a 3-day infection time course. (**C**) Msm isolated from naïve macrophages. (**D**) Msm isolated from IFN-γ–stimulated macrophages. (C) and (D) Infections are representative of three independent experiments. (**E**) Recovery of mean cell stiffness in Msm bacilli isolated from naïve and IFN-γ–stimulated BMDMs, after 72 hours after infection start. Each line represents the trajectory of mean cell surface stiffness for an individual bacillus and is representative of 100 bacilli for ±IFN-γ conditions. (**F**) Schematic representation of the process by which mechanical morphotypes are isolated from macrophages and enriched by buoyancy fractionation. AFM stiffness measurements of mycobacteria isolated from high (<1.064 g/ml) and low (>1.102 g/ml) buoyancy fractions. Horizontal bars represent the mean for each sample. Measurements are representative of three experimental replicates. (**G**) Regrowth quantified from 100 randomly selected mycobacteria isolated after 48 hours of infection. Error bars represent the SEM from three experimental replicates. (**H**) Fractional survival of mycobacteria isolated after 48 hours of infection as compared to infection start. Lateral blue bars represent mean, and dots mean cell surface stiffness measurements from individual bacilli. *P < 0.05 and **P < 0.01 by unpaired *t* test [(C), (D), (F), and (G)] or one-way analysis of variance (ANOVA) (H).

The nonpathogenic Msm represents a technically tractable probe for studying tolerance to stress inflicted by macrophages. We used AFM to measure changes to mean cell surface stiffness of Msm bacilli isolated from bone marrow–derived macrophages (BMDMs) (Fig. 2, A and B). Following infection, individual bacilli predominantly exhibited decreased mean cell surface stiffness (Fig. 2, B and C). Heterogeneity in cell lengths increased over the course of infection in naïve macrophages among mechanically soft bacilli (fig. S6). Some mechanically soft bacilli exhibited elongated chains of uncleaved daughter cells, with two spatially proximal precleavage furrows (Fig. 2B). These chains represent an apparent incapacity for cytokinesed sibling cells to undergo cleavage, a phenotype that is reminiscent of chained peptidoglycan hydrolase mutants or cells incapable of cleavage resulting from reduced turgor pressure (*3*, *4*, *14*). Macrophage stimulation with interferon-γ (IFN-γ) resulted in the emergence of individual bacilli exhibiting increased mean cell surface stiffness (Fig. 2D). Both hard and soft Msm bacilli isolated from macrophages gradually recovered their stiffness to a similar state as axenic conditions of growth (Fig. 2E), verifying that the mechanical changes observed as a result of the intracellular state were reversible upon change of environment. We define these semistable cell states, mechanically differentiated within discrete bounds, as “mechanical morphotypes.”

To expand the study of mechanical properties to larger bacterial populations, AFM results were corroborated using isopycnic centrifugation, which enabled fractionation of bacteria based on buoyancy as a surrogate strategy. Samples were fractionated into three buoyancy states, recovering bacilli in “high” (<1.064 g cm^−3^), “middle” (between 1.064 and 1.102 g cm^−3^), and “low” (>1.102 g cm^−3^) buoyancy fractions, based on reported limits in the fractionation of mycobacterial cultures (*15*). Individuals recovered from a high buoyancy fraction corresponded to increased cell surface stiffness and bacilli recovered from a low buoyancy fraction were mechanically soft by AFM (Fig. 2F). Msm isolated from unstimulated macrophages were predominantly recovered from a low buoyancy fraction and bacilli isolated from IFN-γ–stimulated macrophages were principally recovered from a high buoyancy fraction (figs. S7, A to C), recapitulating our findings using AFM. Bacteria isolated from high and low buoyant fractionations did not exhibit differences in the distributions of cell lengths or elongation velocities (fig. S8). Intracellular Msm isolated from IFN-γ–stimulated macrophages treated with bafilomycin A1, an inhibitor of vacuolar maturation, reversed mechanical morphotype enrichment to a low buoyancy cell state (fig. S9), supporting a role for phagosomal maturation in the selection of the hard mechanical morphotype. Attenuated host macrophage innate immune activity results in recovery of a low buoyancy morphotype, potentially representing a “default” mycobacterial cell state with enhanced survivability.

We evaluated the physiological consequences of mycobacterial differentiation into two distinct mechanical states. Intracellular mycobacteria were isolated from lysed macrophages, fractionated by buoyancy centrifugation (Fig. 2F), and the relative recovery rate was quantified using LTTL-AFM or growth was measured by plated colony-forming units (CFU). Using LTTL-AFM, regrowth of soft bacilli was twofold higher than that of hard bacilli in unstimulated macrophages and threefold higher in IFN-γ–stimulated macrophages (Fig. 2G). Using CFU to quantify the relative survival of low versus high buoyancy fractions, the low buoyancy fraction exhibited 10-fold greater survival than the high buoyancy fraction in unstimulated macrophages and a twofold difference in IFN-stimulated macrophages (Fig. 2H). Mechanisms favoring switching to a soft morphotype afford a selective advantage during infection, even in host macrophage conditions in which the hard mechanical morphotype is selected.

**Fig. 3.**
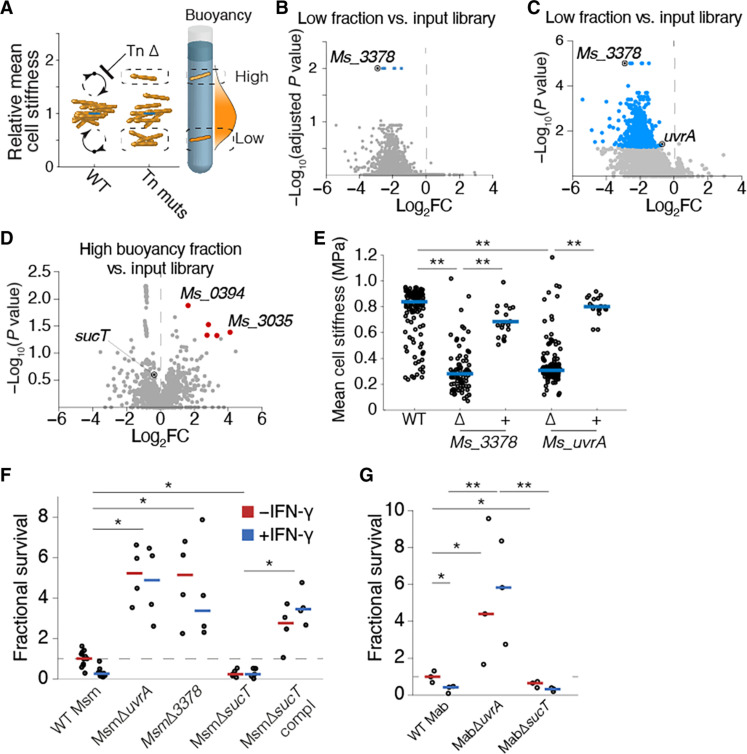
Soft mechanical morphotype mutants have enhanced macrophage survival. (**A**) Schematic representation depicting the selection of mechanically distinct transposon insertion mutants of Msm by buoyancy fractionation. Volcano plot of genes with different transposon insertions against adjusted *P* value (**B**) or *P* value (**C**) by resampling test for TnSeq analysis of low fraction versus input library and high fraction versus input libraries (**D**); genes with differential transposon insertions (see Materials and Methods) are marked in blue and red dots. Black “target” dots in (B) to (D) represent genes selected for further study in Msm and Mab. (**E**) Mean cell surface stiffness quantified using AFM, of WT Msm, soft mechanomorphotype mutants, and complemented strains. Stiffness measurements are representative of two independent experimental replicates (~15 to 60 bacteria counted per condition). (**F** and **G**) Relative recovery of mechanical morphotype mutants in Msm (F) and Mab (G) following a 48-hour infection in macrophages untreated or treated with IFN-γ. Horizontal bars represent means of individual experiments. *P < 0.05 and **P < 0.01 by one-way analysis of variance [(E) to (G)].

These results present the possibility that there exists an order of switching from one semistable mechanical morphotype to another. We hypothesized that mutants shifting the basal mechanical state of Msm into different mechanical morphotypes could be isolated with appropriate selection. High-density transposon-mutagenized libraries in Msm (*16*) were sorted by buoyancy fractionation to identify genes required for regulating the distribution of mechanical cell states in axenic conditions of growth (Fig. 3A). Transposon insertion site frequencies were mapped by sequencing (TnSeq) and compared with the unfractionated input library. Conditional essentiality of transposon mutants was identified by the absence of transposon insertions found in low (soft) or high (hard) buoyancy fractions. These corresponded to mutants for which the capacity to actively switch out of soft or hard mechanical morphotypes in standard axenic culture was compromised, resulting in enrichment of the differentiated morphotype at a basal mechanical cell state. We identified a discrete group of genes negatively regulating the low buoyancy mechanical morphotype (Fig. 3B). None of the hits identified following stringent post hoc correction was among highly conserved genes, potentially due to the saprophytic nature of Msm. To extend our findings to other mycobacteria, we asked whether relaxing the post hoc correction would enable us to identify conserved conditionally essential genes for each discrete mechanical morphotype. We identified many more mutants that appeared to be exclusively enriched in a low buoyancy fraction. By contrast, very few mutants were identified whose basal mechanical cell state was shifted into a high buoyancy fraction (Figs. 3, C and D). The top candidate identified among the low-buoyancy mechanomorphotype mutants was ∆*Ms3378*, a predicted β-lactamase (Fig. 3B). Another candidate gene identified in our screen, *uvrA*, is characterized as a member of the *uvrABC* gene system responsible for DNA damage repair and widely conserved in bacteria (Fig. 3C) (*17*, *18*). Both ∆*Ms3378* and ∆*uvrA* exhibited decreased cell surface stiffness by AFM as compared to wild-type (WT) Msm (Fig. 3E and fig. S10), confirming the validity of our screening approach.

Buoyancy fractionation enabled us to evaluate the generalizability of mechanical morphotype switching in pathogenic bacteria, which was not possible by AFM due to lack of an instrument in biosafety containment. We interrogated the distribution in mechanical cell states of Mab, a nontuberculous mycobacterium and pathogen. Mab from axenic culture was enriched mostly in the low buoyancy fraction (fig. S8D), representing an important shift as compared with Msm. Smooth and rough colony morphology variants (*19*) did not influence distributions in buoyancy (figs. S7, G and H). Mab recovered from macrophages exhibited the same distributions in buoyancy fractionation as Msm: Low buoyancy fractions predominated in unstimulated macrophages and high buoyancy fractions predominated in IFN-γ–stimulated macrophages (figs. S7, E and F), supporting the generalizability of our findings to both nonpathogenic and pathogenic mycobacteria.

We chose *sucT* to evaluate the survival of a mutant representing a hard mechanomorphotype, a mutant exhibiting elevated mean cell surface stiffness (*13*), despite having identified that ∆*sucT* was not identified in our post hoc analysis of the TnSeq analysis. We confirmed that ∆*uvrA* and ∆*sucT* had similar phenotypes in Msm and Mab. Buoyancy fractionation of Msm-∆*sucT* and Mab-∆*sucT* led to recovery of bacteria predominantly from high buoyancy fractions, and this phenotype could be complemented genetically (figs. S11 and S12). As with Msm, Mab-∆*uvrA* was isolated mostly from low buoyancy fractions (fig. S13), confirming that these mutations influence the mechanical state of the cell. Mab-∆*uvrA* was enriched in a low buoyancy cell state when isolated from unstimulated macrophages; however, IFN-γ–stimulated macrophages drove a shift in the basal mechanical cell state. Mab-∆*sucT* was recovered predominantly from a high buoyancy fraction in both unstimulated and IFN-γ–stimulated macrophages (fig. S13).

Do mechanical morphotype mutants show altered survival during macrophage infection? The soft mechanomorphotype mutants, Msm-∆*uvrA* and Msm-∆*Ms3378*, both exhibited increased survival in macrophages, particularly in IFN-γ–stimulated macrophages with a ~6-fold and >20-fold increase, respectively, as compared to WT (Fig. 3F). Survival of Msm-∆*sucT* was attenuated compared with WT in both unstimulated and IFN-γ–stimulated macrophages (Fig. 3F). Similarly, Mab-Δ*sucT* had twofold decreased survival during infection of unstimulated macrophages as compared with WT, a host cell condition selecting for the predominance of the soft mechanical state (Fig. 3G). In IFN-γ–stimulated macrophages, which drive selection of a hard mechanical state (fig. S7F), WT Mab and Mab-Δ*sucT* had similar rates of recovery (Fig. 3G). By contrast, Mab-Δ*uvrA* isolated from unstimulated macrophages had 20% increased survival as compared with WT bacteria, and >5-fold increased survival in the context of IFN-γ stimulation (Fig. 3G). No differences in elongation dynamics were observed between the mechanical morphotype mutants and WT Msm bacilli (fig. S8C).

**Fig. 4.**
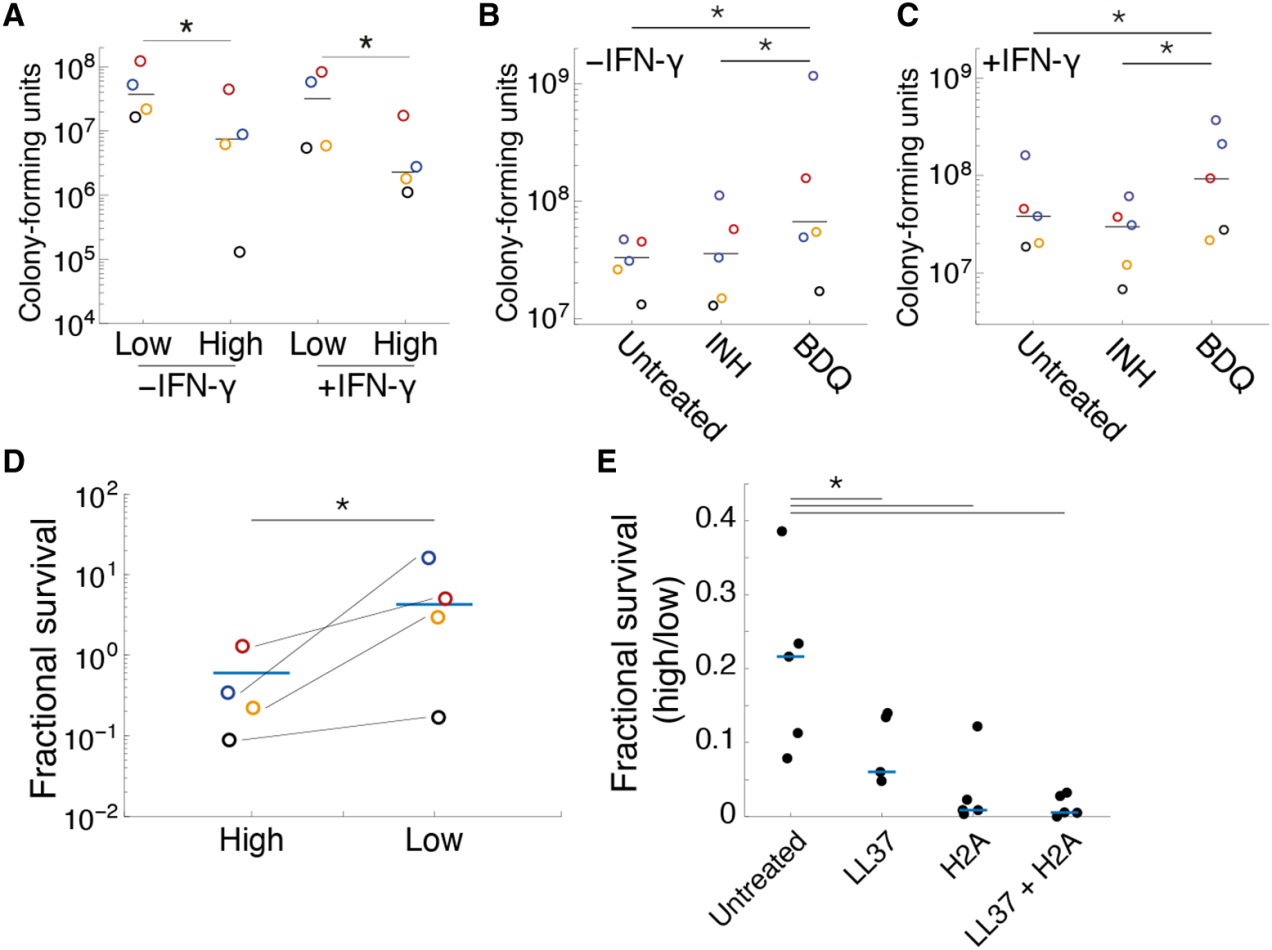
Phenotypic conditioning of mechanical state alters survival of Mab to macrophage-derived stress. Mab was pre-sorted by buoyancy fractionation (**A**) or preconditioned by sub-MIC antibiotic treatment (**B** and **C**) into high-buoyancy (hard) and low-buoyancy (soft) mechanical morphotypes and then used to infect macrophages (MOI = 10 bacteria per macrophage) for 48 hours. Survival was quantified by harvesting bacteria from macrophages and quantifying CFU. (**D**) Mechanical morphotypes in Mab, isolated by buoyancy fractionation, were subjected to the antimicrobial peptide, LL37. Fractional survival of LL37-treated (5 μg/ml) versus untreated prefractionated Mab mechanical morphotypes. Each colored dot represents a separate experiment, and bars represent the means of the experimental replicates. (**E**) Mab was treated for 2 hours with LL37 (1 μg/ml), histone H2A (500 μg/ml), and a combination of both, before mechanical morphotypes were isolated by buoyancy fractionation. A ratio of fractional survival of high versus low buoyancy fractions illustrates decreased survival of the high buoyancy fraction in treatments with H2A. Each point is representative of an individual experiment. *P < 0.05 by unpaired *t* test [(A) and (D)] or one-way analysis of variance [(B), (C), and (E)].

We had demonstrated that genetic mutants that drive distinct mechanical morphotype states could influence mycobacterial survival within macrophages. Next, we wished to ask whether altering the mechanical state of fully WT Mab could affect survival in macrophages. Macrophages were infected with WT Mab prefractionated into high and low buoyancy fractions. Low buoyancy fractions of Mab had ~5-fold increased survival in unstimulated macrophages and >10-fold increased survival in IFN-γ–stimulated macrophages, as compared with prefractionated high buoyancy morphotypes (Fig. 4A). In an orthogonal approach to affect Mab mechanical state, we used subinhibitory concentrations of antibiotics to precondition WT Mab. INH was used to produce hard bacilli and BDQ was used to generate soft mechanical morphotype bacilli (fig. S3, A and C). BDQ-conditioned bacilli exhibited ~2-fold increased survival in unstimulated macrophages and greater than 5-fold increased survival in IFN-γ–stimulated macrophages as compared to both untreated and INH-conditioned bacilli (Fig. 4, B and C). Our results suggest that the impact of antibiotics on mechanical morphotype switching and, hence, macrophage-mediated survival may be an important consideration in treating mycobacterial infections.

The antimicrobial peptide cathelicidin (LL37) contributes to macrophage-mediated killing of intracellular pathogens, including mycobacteria (*20*) through physical damage. Do mechanical morphotype mutants exhibit differences in survival to LL37 treatment? WT Mab was prefractionated by buoyancy centrifugation into low and high buoyancy fractions and subsequently treated with LL37. The low buoyancy fraction exhibited ~7-fold increased viability to LL37 treatment as compared to the high buoyancy fraction (Fig. 4D). Emerging evidence suggests that LL37 in synergy with histones provide increased bacterial killing (*21*). Treatments with LL37 and Histone H2A drive both selective killing and mechanical morphotype switching (fig. S13). Histone H2A treatment was sufficient to provoke a 10-fold relative decrease in the high buoyancy fraction as compared to the low buoyancy fraction (Fig. 4E), suggesting that H2A selectively reduces the relative numbers of Mab in a high-buoyancy cell state. H2A treatment equally drove an increase in the numbers of Mab recovered from the low buoyancy fraction as compared to an untreated Mab (fig. S14), suggesting that the soft mechanical morphotype exhibits increased tolerance and antimicrobial peptide treatments provoke mechanical morphotype switching. These results suggest that the low buoyancy fraction (soft mechanical morphotype) represents an innate immune stress-tolerant phenotypic state in mycobacteria.

## DISCUSSION

Previous studies outline limits in the mechanical variation of bacterial cell wall integrity and cytosolic tonicity (*2*, *15*, *22*–*24*), both of which are fundamental mechanical cell properties influencing the impact of bactericidal stress. Divergent trajectories of mechanical morphotype switching to hard and soft cell states raise the possibility for bacilli to actively respond to diverse and distinct stresses. The host drivers of selection and microbial molecular regulators of mechanical morphotype enrichment together represent a new basis for host-pathogen interactions.

How is mechanical morphotype switching controlled? We demonstrate that mycobacterial “mechanical morphotype switching” requires the modification of physical cell properties, represented by cell wall integrity and turgor pressure. The timescale of switching highlights the predominant physical cell properties capable of driving the trajectory of mechanical morphotype switching. Turgor pressure is a rapidly modulated bacterial cell property—on a subsecond timescale—driving cell wall deformation (*2*, *25*). We demonstrate that turgor pressure is important for switching mycobacterial bacilli into a soft mechanical morphotype within minutes. The control of mycobacterial turgor pressure is likely a critical regulator of switching to a soft mechanical morphotype state, with downstream phenotypic impact for intracellular mycobacterial cells.

In contrast to the rapid switching to the soft mechanical morphotype, the hard mechanical morphotype emerges gradually over the course of hours. Molecular perturbation of outer cell wall biosynthetic processes results in an increased mean cell surface stiffness on mycobacterial cells (*1*, *12*, *13*). Why might outer cell wall perturbation drive emergence of a hard mechanical morphotype? Perturbation of the mycolate layer results in a “thinning” of the outer cell wall layer, potentially enabling the AFM to probe a more load-bearing, sugar-rich inner cell wall layer, the peptidoglycan (*1*, *12*, *13*). Reduced cell wall thickness increases strain hardening deformation of the remaining cell wall. Managing the architecture of the cell wall is likely critical for managing physicochemical environmental fluctuations for bacteria to prevent mechanical failure.

It is possible that other physiological cell processes control switching into mechanically differentiated morphotypes. Growth in hypoxic conditions is reported to influence mycobacterial cell wall biosynthesis and induce lipid inclusions (*26*, *27*), culminating in an expanded distribution in buoyant density of *M. tuberculosis* as compared to aerobic conditions (*15*).

The basis for mechanical adaptation includes active molecular control of the mechanical cell state to enhance survival during macrophage infection. We identified molecular factors controlling the enrichment of discrete mechanical morphotypes, revealing molecular mechanisms actively limiting mechanical differentiation as opposed to drivers of switching. Redundant control mechanisms or the lack of positive regulation may account for why we did not identify any transcription factor(s) controlling “switching.” Alternatively, a positive regulator may be an essential gene that would not be identifiable by our forward genetic screen. Our results suggest that mycobacteria continuously shuttle through a soft mechanical morphotype state and that molecular factors actively enable bacteria to switch back into a “moderate” mechanical morphotype. This molecular process constrains phenotypic heterogeneity in axenic conditions of growth and enables differentiation of the basal mechanical cell state when encountering a variety of stresses. By contrast, few genetic mutants were identified modifying the basal mechanical cell state to the hard mechanical morphotype. It is possible that bacteria do not spontaneously cycle through a hard mechanical morphotype in axenic culture, rather requiring a trigger to provoke switching, which host intracellular conditions provide. These mechanisms controlling mechanical morphotype switching are reminiscent of the proposed mechanisms underlying the emergence of phenotypically resistant “persister” cells (*28*).

What are the biological implications of mechanical morphotype switching for mycobacteria? During infection, soft mechanical morphotype individuals emerge as chains exhibiting double septa near mid-cell, likely due to defective cleavage of cytokinesed sibling cells. Turgor pressure, a hallmark of soft mechanical morphotype cells, is necessary for driving cleavage of cytokinesed daughter cells, resulting in chaining (*4*). Chained mycobacteria are largely viable (*14*), though exhibit restricted unipolar growth dynamics resulting from inhibition of elongation at the chained new poles (*3*, *7*). Increased survival of the soft mechanical morphotype in macrophages suggests that division defects are tolerated under restrictive host innate immune selection. Increased tolerance of the soft mechanical morphotype might result from decreased cell permeability and accumulation of small molecules (*12*). In contrast, the hard mechanical morphotype exhibits reduced survival resulting from more restrictive host innate immune pressures. How might IFN stimulation enhance intracellular mycobacterial clearance? Increased stiffness resulting from a reduced mycolate layer is correlated with increased permeability and cytosolic accumulation of hydrophilic molecules (*12*). Lysosomal fusion with *Mycobacterium*-containing vacuoles restricts infection and perturbs mycobacterial cell wall integrity (*29*). While recovery from switching to a hard mechanical morphotype is observed, we cannot rule out that this transition among nonsurvivors is unidirectional and terminal, in activated macrophages.

The long coevolutionary interaction between human host cells and mycobacterial pathogens has likely resulted in the emergence of compensatory mechanisms in mycobacteria enabling for intracellular bacilli to tolerate environments driving cells into increased stiffness regimes. A mechanical basis for host-pathogen interactions plays on host cells driving the stress-strain relationship on the bacterial cell past the ultimate tensile strength toward the failure point, while bacterial pathogens aim to maintain physical properties near the yield strength limit and attenuating the impact of strain hardening that the cell wall withstands.

The combination of bacterial intrinsic and extrinsic (host) factors influencing the emergence of discrete mechanical morphotypes highlight a new basis for studying host-pathogen interactions. We identified genes influencing mycobacterial cell surface mechanical properties that are highly conserved in evolutionarily disparate prokaryotes. Microbial cell mechanical morphotype switching may represent a fundamental cell process in all bacteria harnessed to respond to changing environmental conditions. Our findings fundamentally expand the understanding of mycobacterial virulence to include mechanical adaptation as an important driver of survival during infection.

## MATERIALS AND METHODS

### Bacteria

Msm mc^2^155 (WT) and derivative strains and Mab [American Type Culture Collection (ATCC) 19977] and derivative strains were grown in Middlebrook 7H9 liquid medium (Difco) supplemented with 0.5% albumin, 0.2% glucose, 0.085% NaCl, 0.5% glycerol, and 0.5% Tween-80. Cultures were grown at 37°C to mid-exponential phase [optical density at 600 nm (OD_600_) of ~0.5]. Aliquots were stored in 15% glycerol at −80°C and thawed at room temperature before use. The ∆*uvrA* strains were made by allelic exchange with an unmarked in-frame deletion of the *uvrA* gene in both Msm and Mab. The *attB*-integrating plasmid expressing a *uvrA*-*wasabi* fusion was used to complement the gene *uvrA* into the *Ms*∆*uvrA* strain. Construction of the Msm *sucT* complementation strain was described previously (*13*). Chemicomechanical manipulation of mycobacteria was conducted by treatments with INH (Sigma-Aldrich) at 10 μg ml^−1^ [2× minimum inhibitory concentration (MIC)] or 5 μM CCCP, cathelicidin (LL37; Thermo Fisher Scientific) at 0.1 and 0.5 μg ml^−1^, and histone H2A (Thermo Fisher Scientific) at 500 μg ml^−1^.

#### Mab ATCC 19977 uvrA KO

Deletion of *uvrA* (*MAB_2315*) from Mab ATCC 19977 was carried out using an ORBIT system described by Murphy *et al.* (*30*). Briefly, Mab was transformed with plasmid pKM444 expressing the Che9c phage RecT annealase and the Bxb1 phage integrase under control of the inducible pTet promoter. A 20-ml culture of *Mabs* pKM444 was grown at 37°C until OD = 0.5, anhydrotetracycline was added at a final concentration of 500 ng/ml to induce expression, and cells were further incubated for 4 hours until OD = 1. At this point, cells were harvested, washed three times with 10% glycerol, and resuspended in 2 ml of 10% glycerol. An aliquot of 380 ml of cells was cotransformed with 200 ng of payload plasmid pKM496 (Zeo^R^) plus 1 mg of ORBIT oligo and incubated overnight with shaking at 37°C to let them recover. Cells were collected and spread on 7H11 albumin-dextrose-catalase (ADC) + Zeo (100 mg/ml); plates were incubated for 5 days at 37°C, and colonies were picked and checked by polymerase chain reaction (PCR) to verify the recombinants.

### Transposon library construction, genomic DNA extraction, and sequencing

Transposon library after buoyancy centrifugation was collected and resuspended in 400 μl of 10 mM tris (pH 9). After bead beating, genomic DNA was extracted by the phenol-chloroform method. DNA concentration was measured and quantified by NanoDrop and Qubit. For building a transposon sequencing library, approximately 5 μg of genomic DNA was resuspended in 150 μl of Tris-EDTA (TE) buffer and transferred to a Covaris tube, and genomic DNA was disrupted to 200– to 500–base pair size range by sonication with the following parameters: duty cycle (10%), intensity (4), cycles/burst (200), time (80 s). The fragmented genomic DNA was size-selected and purified by AMPure XP beads. The fragmented genomic DNA was further subjected to end repair and dA tailing. Annealed adapter was ligated to the dA-tailed fragmented genomic DNA, and the linked ligated DNA fragment was used as template of the first-round nested PCR to amplify fragments containing adaptor and transposon junction. Indexed barcoded sequencing and Illumina sequencing adaptor was added by the second nested PCR. All sequence libraries were examined by Agilent 2100 Bioanalyzer and subjected to next-generation sequencing.

### Transposon mutagenesis

Msm mc^2^155 strain was grown to stationary phase (OD > 6) in 50 ml of 7H9 growth medium. Bacterial cultures were washed and resuspended in 5 ml of mycobacteriophage (MP) buffer (50 mM tris, 150 mM NaCl, 10 mM MgSO_4_, and 2 mM CaCl_2_). To transduce bacteria with MycoMarT7 phage, approximately 10^11^ plaque-forming units of phage was added to the bacterial suspension in MP buffer and incubated at 37°C for 4 hours. Immediately after transduction, ~300 to 400 μl of the transduction mixture was plated on 15-cm LB agar plates, containing kanamycin (20 μg/ml) and 0.1% Tween-80. After 3 days, library size was determined, and bacteria were scrapped and stored in 7H9 medium plus 15% glycerol as library stock. The transposon library was made in triplicate. The transposon library was cultured to an OD_600 nm_ of 0.8, and 1 ml of sample was loaded onto 10 ml of stock isotonic Percoll medium, with buoyant density beads as fiducial markers. Buoyancy centrifugation was conducted at 18°C and spinning at 20,000 rpm (~50,000*g*) for 1 hour and 20 min. Three buoyancy fractions were isolated: high (>1.02, <1.064 g cm^−3^), middle (>1.064, <1.102 g cm^−3^), and low (>1.102 g cm^−3^). Three biological replicates of the buoyancy centrifugation were conducted for the transposon library made in triplicate each of the three transposon libraries: 3 (libraries) × 3 (buoyancy centrifugation experiments) × 3 (buoyancy fractions) = 27 individual samples.

### Transposon mapping and analysis

Reads processing and TA loci mapping were performed through software TRANSIT (*31*). Loci that were differentially disrupted by transposon were analyzed using resampling test in TRANSIT with a default parameter. Different buoyancy fractions were compared with input libraries, and genes that are overrepresented [log_2_ fold change (log_2_FC) ≤ −1, adjusted *P* value of <0.05] and underrepresented (log_2_FC > 1, *P* value of <0.05) were plotted.

### ORBIT oligo for uvrA KO

The oligonucleotide sequence used for deletion of uvrA was as follows: CGGTTCACCAACGGCGGCGTCAGTCATGACTGCCACCCTAGACCGGAGTGACAACCTTCCTGGTCCGCGCGGTTTGTACCGTACACCACTGAGACCGCGGTGGTTGACCAGACAAACCCGCGCCCCGCACGATCAGACGGTCGGCCACCGGTCTCCTTTCACACTGCCCTATGCAGGTGTTTTCGCGT.

### Cell culture and infection

BMDMs were differentiated from cryopreserved bone marrow stocks extracted from femurs of 8-week-old C57BL/6 mice. After cultivation for 7 days in petri dishes in BMDM differentiation medium [Dulbecco’s modified Eagle’s medium (DMEM) with 10% fetal bovine serum (FBS), 1% sodium pyruvate, 1% GlutaMAX, and 20% L929 cell–conditioned medium as a source of granulocyte/macrophage colony-stimulating factor], adherent cells were gently lifted from the plate using a cell scraper, resuspended in BMDM culture medium (DMEM with 5% FBS, 1% sodium pyruvate, 1% GlutaMAX, and 5% L929-cell–conditioned medium), and seeded on the plate used for the experiment. For infection, 1 ml of Msm or Mab culture at an OD_600_ of 0.4 to 0.8 was pelleted, resuspended in 200 μl of BMDM culture medium, and passed through a 5-μm filter to eliminate bacterial aggregates. The resulting single-cell suspension was used to infect BMDMs at a multiplicity of infection (MOI) of 1:1. After 4 hours of infection, macrophages were washed extensively to remove extracellular bacteria and incubated with fresh macrophage medium. All the incubations were performed at 37°C, 5% CO_2_. When required, IFN-γ (100 U/ml) was added to the macrophage medium 16 hours before infection and kept during the experiment. At selected time points, postinfection macrophages were lysed with 0.5% Triton X-100 in phosphate-buffered saline (PBS) for 5 min to collect the intracellular bacteria. The cell lysate was pelleted and washed twice with PBS, and the pellet was resuspended in 7H9 for further microscope imaging.

RAW264.7 macrophages were infected with Mab or Msm (MOI 10:1) for 30 min before washing cells and adding gentamicin (50 μg/ml) for 1 hour to remove extracellular bacilli. Serial dilutions of MOI-adjusted bacterial input were plated to quantify CFU and subsequently used to control for variations in MOI. When required, IFN-γ (100 U/ml) was added to macrophages 12 hours before infection and kept during the experiment. At 48 hours of infection, macrophages were thoroughly washed with fresh medium and lysed with 0.5% Triton X-100 in PBS for 5 min to collect intracellular bacilli. The cell lysate was pelleted and washed twice with PBS to remove detergent, and samples were either serially diluted to plate for CFU or loaded onto standard isotonic Percoll to fractionate differentially buoyant bacilli by buoyancy gradient centrifugation.

### Optical fluorescence microscopy

Bacteria extracted from macrophages were seeded on a 35-mm Ibidi μ-dish and imaged for up to 24 hours at 1-hour intervals to check for growth recovery. Infected BMDMs were imaged for up to 72 hours at 1-hour intervals. All the microscopy images were acquired on a DeltaVision microscope equipped with an enclosure maintaining the temperature of the sample at 37°C, fluorescein isothiocyanate (excitation: 490/20 and emission: 525/36) and tetramethyl rhodamine isothiocyanate (excitation: 555/25 and emission: 605/52) filters and 60× or 100× objectives. Host cells and bacteria were respectively identified in bright-field and fluorescence images. Multiple XY fields were acquired in parallel. For microscope experiments with macrophages, samples were maintained in a humidified stage-top incubator connected to a gas mixer (Okolab) supplying air mixed to 5% CO_2_. For some experiments, macrophages imaged by time-lapse microscopy were lysed by replacing the macrophage medium with 0.5% Triton X-100 in PBS using custom-made tubing connected to the lid of the macrophage dish. After 5 min, cells were washed gently with PBS and then incubated with 7H9 medium supplemented with calcein-AM (100 ng ml^−1^) to identify live, enzymatically active cells. Bacteria sticking to the debris of the lysed cells were imaged by time-lapse microscopy to track their regrowth.

### Image analysis

The microscopy images and time series were analyzed using the Fiji software from the ImageJ package (*32*). Macrophage infection status and viability were monitored by visual analysis of the time-lapse bright-field image series. Regions of interest corresponding to individual macrophages were manually drawn onto the bright-field images and transferred to fluorescence images. A manual threshold was set on the fluorescent channel to segment the bacteria. The area above the threshold for each region of interest was measured for each time point and used as a proxy for the number of intracellular bacteria per cell. The bacterial growth rate of each individual intracellular microcolony was calculated by fitting an exponential curve to the measured fluorescent areas. For individual bacteria extracted from lysed macrophages, regrowth and staining were manually analyzed through visual inspection of the microscope image series.

### AFM imaging

Coverslips were prepared as previously described (*3*). Polydimethylsiloxane (PDMS; Sylgard 184, Dow Corning) at a ratio of 15:1 (elastomer:curing agent) was cut 1:10 with hexane to reduce the spin-coated layer 10-fold while equally increasing the hydrophobicity of the surface. Aliquots of mycobacteria isolated from axenic culture or from infection of macrophages were filtered through a 0.5-μm-pore-size polyvinylidene difluoride filter (Millipore) to remove cell clumps and enrich single cells. Aliquots were deposited on the hydrophobic surface of a PDMS-coated coverslip. 7H9 growth medium was supplied. Where indicated, antibiotic was added to the growth medium. The medium was maintained at 37°C using a custom-made heating element within the sample space and a TC2-80-150 temperature controller (Bioscience Tools). Bacteria were imaged by peak force tapping using a Nanoscope 5 controller (Veeco Metrology) at a scan rate of 0.25 to 0.5 Hz and a maximum *z* range of 5 μm. A ScanAsyst fluid cantilever (Bruker) was used. Continuous scanning provided snapshots at 2- to 30-min intervals. Height, peak force error, adhesion, dissipation, deformation modulus, and log modulus were recorded for all scanned images. Peak force error yields a fine representation of the height on the order of 10 nm in the *z* axis; this is computed as the difference between the peak force set point and the actual value. Images were processed using Gwyddion (Department of Nanometrology, Czech Metrology Institute). ImageJ was used for extracting bacterial cell surface height and modulus values and generating dynamic and quantitative mean values of the mechanical properties of individual cells.

### Correlated fluorescence and AFM

Correlated fluorescence and AFM images were acquired as described previously (*3*, *33*). Briefly, fluorescence images were acquired with an electron-multiplying charge-coupled device (EMCCD) iXon Ultra 897 camera (Andor) mounted on an IX71 inverted optical microscope (Olympus) equipped with an UAPON100XOTIRF 100× oil immersion objective (Olympus) with the ×2 magnifier in place. Illumination was provided by a monolithic laser combiner (Agilent) using the 488- or 561-nm laser output coupled to an optical fiber with appropriate filter sets: F36-526 for calcein-AM and F71-866 for mCherry-Wag31 or cytosolic red fluorescent protein. The AFM was mounted on top of the inverted microscope, and images were acquired with a customized Icon scan head (Bruker) using ScanAsyst fluid cantilevers (Bruker) with a nominal spring constant of 0.7 N m^−1^ in peak force tapping mode at a set point of <2 nN and typical scan rates of 0.5 Hz. The samples were maintained at 37°C in 7H9 growth medium heated by a custom-made coverslip heating holder controlled by a TC2-80-150 temperature controller (Bioscience Tools).

### Cell measurements

Fundamental physiological cell measurements of both cell dimensions and mechanical surface rigidity were made as described previously (*1*, *3*, *4*, *12*, *13*).

#### Cell growth measurements

Cell growth herein describes a process of cell expansion, which is defined as a function of both cell elongation along the long axis of the cell and surface mechanical maturation (change in cell surface stiffness) upon the addition of nascent cell wall material. Cell length was measured as per (*3*, *4*, *7*). Mechanically distinct cell growth zones were segregated into three spatially discrete zones based on the dynamic and mechanical state of the surface: (i) The nascent growth zone consists of mechanically soft material typically localized nearest the poles in mycobacteria, (ii) a maturation zone consists of the space adjacent to the nascent zone where surface material is dynamically increasing in mechanical rigidity, and (iii) a mechanically mature zone where surface material mechanical properties has ceased to increase.

#### Mean cell surface stiffness

Mycobacterial cell surface stiffness is averaged over the relatively flat surface along the longitudinal midline of the cell surface, which is probed by AFM imaging in peak force tapping mode and interpreted as the Young’s modulus as per the Derjaguin-Muller-Toporov (DMT) model. The absolute values can vary from one experiment to the next, as a function of variations in the biophysical properties of the fluid medium (temperature and fluid density). Therefore, the mean cell surface rigidity is interpreted as a relative measure comparing the bacterium to the modulus of the PDMS-coated coverslip sample surface (fig. S15).

#### Buoyant density

Buoyancy fractionation was conducted by centrifugation of mycobacteria at high speeds in a viscous, gradient-forming medium. Stock isotonic Percoll medium (SIP) is prepared by mixing 9 ml of Percoll with 1 ml of 0.15 M NaCl. Ten milliliters of SIP was loaded into thin-walled polypropylene centrifuge tubes (14 × 89 mm) (Beckman Coulter) along with 1 ml of bacterial sample and 50 μl of buoyancy centrifuge beads. Mycobacteria were centrifuged at 20,000 rpm (~52,000*g*) for 1 hour and 20 min using a Beckman Coulter Optima L-90K Ultracentrifuge and a SW40Ti swinging bucket rotor. Buoyant density beads (Cospheric) at 1.02, 1.064, 1.08, and 1.102 g cm^−3^ were used to distinguish high, middle, and low buoyancy fractions of mycobacteria.
