# Supplementary material for: Mechanical morphotype switching as an adaptive response in mycobacteria
Source: Sci Adv. 2024 Jan 3;10(1):eadh7957. doi: 10.1126/sciadv.adh7957 (PMC10776010; doi:10.1126/sciadv.adh7957)
Supplement: Supplementary file 1 — Figs. S1 to S15 Legends for movies S1 to S4 [file sciadv.adh7957_sm.pdf]

Science Advances
AAAS

Supplementary Materials for

Mechanical morphotype switching as an adaptive response in mycobacteria

Haig Alexander Eskandarian *et al.*

Corresponding author: Haig Alexander Eskandarian, haeskandarian@gmail.com; Babak Javid, babak.javid@ucsf.edu

Sci. Adv. **10**, eadh7957 (2024)
DOI: 10.1126/sciadv.adh7957

The PDF file includes:

Figs. S1 to S15
Legends for movies S1 to S4

Other Supplementary Material for this manuscript includes the following:

Movies S1 to S4

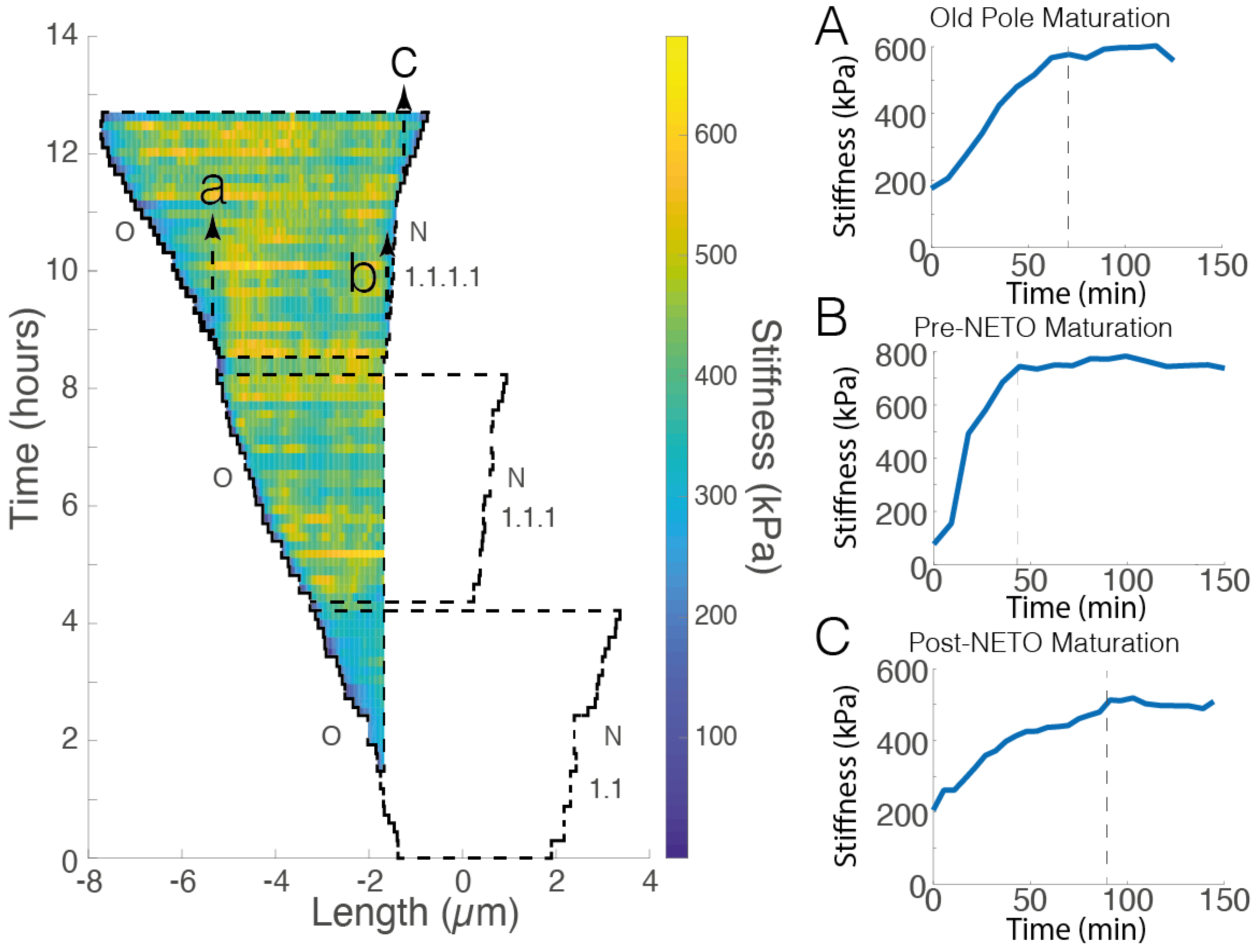

Fig. S1. Mycobacterial cell surface mechanical growth dynamics.
Kymograph representation of the cell surface of *M. smegmatis* over three successive generations of growth and division. Depicted is the spatial inheritance of cell surface material from when it is first deposited as a result of polar elongation. **(A – C)**, The dynamic of "mechanical maturation" of nascent material increases in surface stiffness until it plateaus. The rate of maturation is distinguishable depending on the age of the pole and the timing of "new-end take-off" at the new pole (*7*). The spatial mobility of mechanically mature material is static, as is equally reported for chemically distinct layers (*33*).

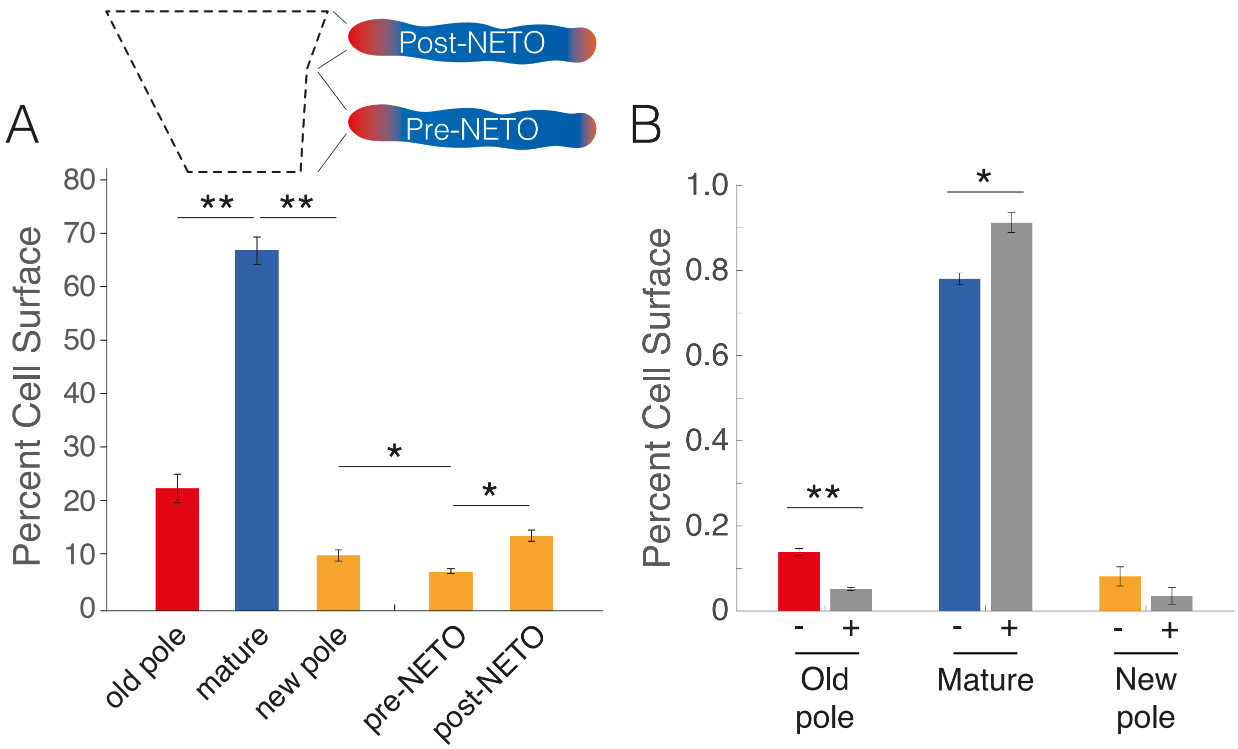

Fig. S2. The spatial percentage of the cell surface of *M. smegmatis* as measured by LTTL-AFM quantitative nanomechanical mapping. (A) In growing *M. smegmatis* in axenic conditions of growth, mechanically stable cell surface material represents >60% of the cell surface (blue bar). Cell surface material that is actively undergoing an increase in mechanical rigidity represents ~30% of the cell surface split between the old and new poles (red and orange bars). The spatial representation of new pole material can be distinguished as a function of whether new-end take-off (NETO) has taken place (*7*). **(B)** Concomitant with cessation of elongation, INH-treated bacilli (grey bars) exhibit decreased spatial distributions of mechanically nascent cell surface material at each of the cell poles. Bars represent mean +/- SEM. *$P < 0.05$, **$P < 0.01$ by an unpaired T-test.

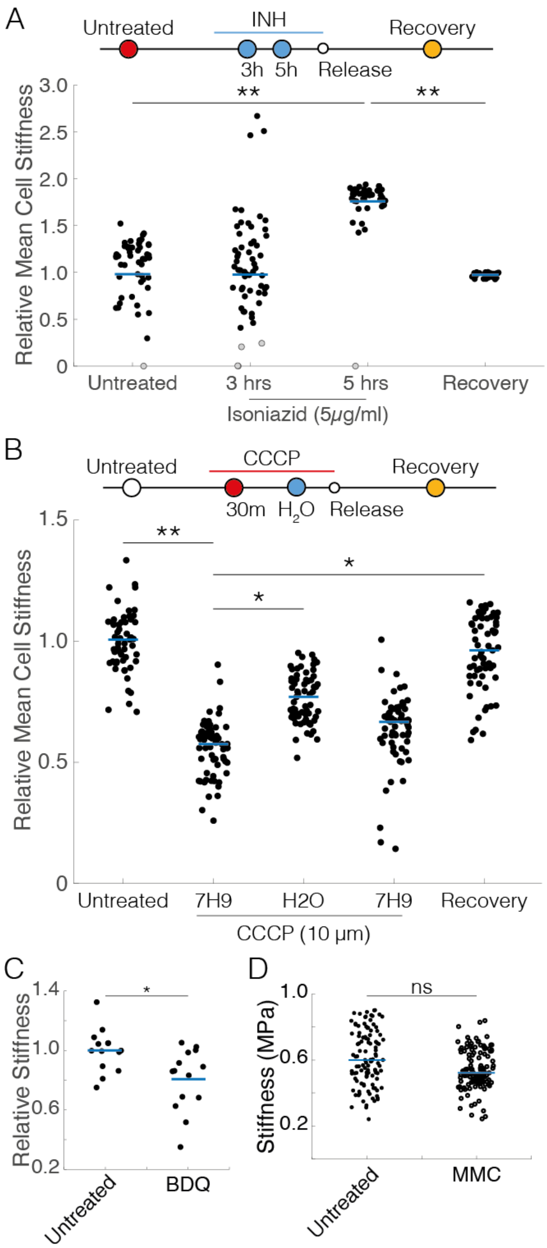

Fig. S3. Relative mean cell surface stiffness of antibiotic-treated *M. smegmatis*.

M. smegmatis treated with INH (5 μg/ml) **(A)**, CCCP (10 μM) **(B)**, BDQ (0.2 μM) **(C)**, or mitomycin C (MMC 50 ng ml^{-1}) **(D)**. Bacilli treated with CCCP was probed within 30 minutes; CCCP-treated bacilli were subsequently exposed to osmotic shock by transiently exchanging growth medium (7H9) with dH_2O, before adding back 7H9 with CCCP, before later releasing bacilli from CCCP-treatment. Recovery represents the mean cell surface stiffness of cells surviving antibiotic treatment, which undergo regrowth and culminate in division, at which point the cell stiffness is probed. Bacilli treated with BDQ were quantified for their change in surface stiffness within 30 minutes and result in a >20% decrease in mean cell surface stiffness. The relative mean cell surface stiffness represents a comparison of the mean cell surface stiffness for treated versus untreated cells. Black dots represent living cells. Mean cell stiffness measurements are representative of three independent experimental replicates. Grey dots represent dead cells **(A)**. Per condition, ~20 – 80 bacteria counted (A & B), ~10 – 20 bacteria (C), and ~100 (D). $*P < 0.05$, $**P < 0.01$ by Mann-Whitney U test.

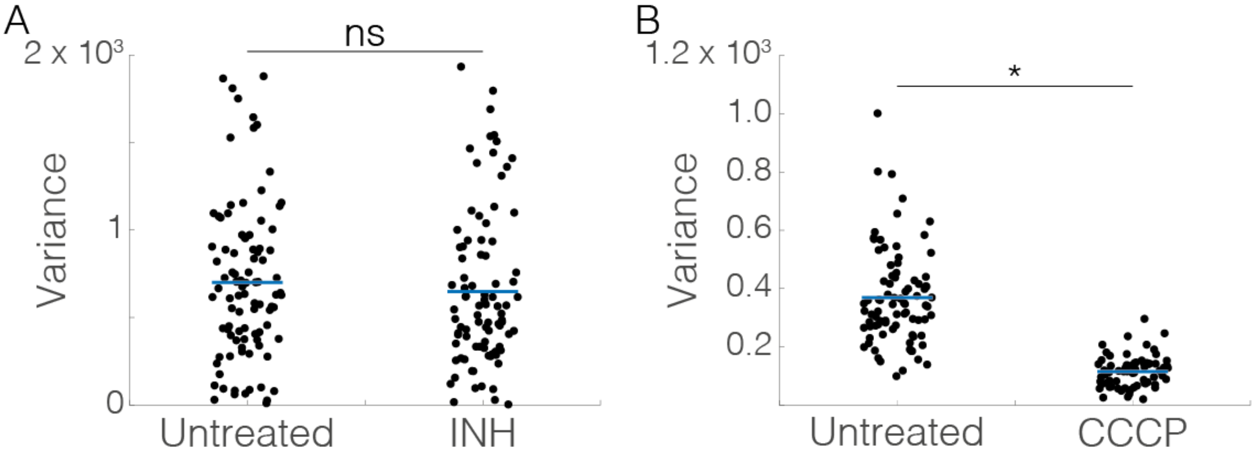

Fig. S4. Variance in mycobacterial cell surface stiffness.

M. smegmatis bacilli, treated with CCCP (a) or INH (b) are quantified for the variation in cell surface stiffness over its full length. Measurements are representative of three independent experimental replicates. ~70 – 100 bacteria counted per condition. *$P < 0.05$ by unpaired T-test.

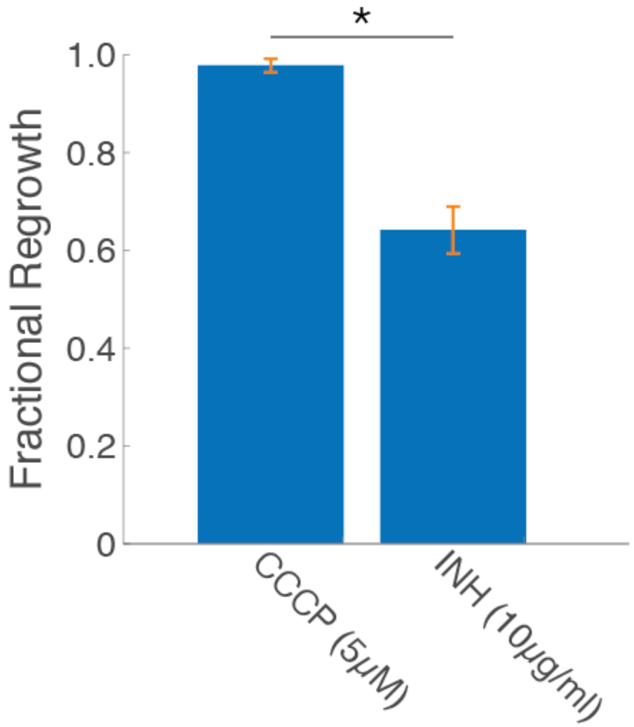

Fig. S5. Fractional recovery of *M. smegmatis* released from antibiotic treatment.
M. smegmatis bacilli, released from antibiotic treatment during LTTL-AFM imaging at 37°C, are counted for their ability to regrow among all surviving bacteria. Horizontal bars represent mean +/- SEM. $*P < 0.05$ by unpaired T-test.

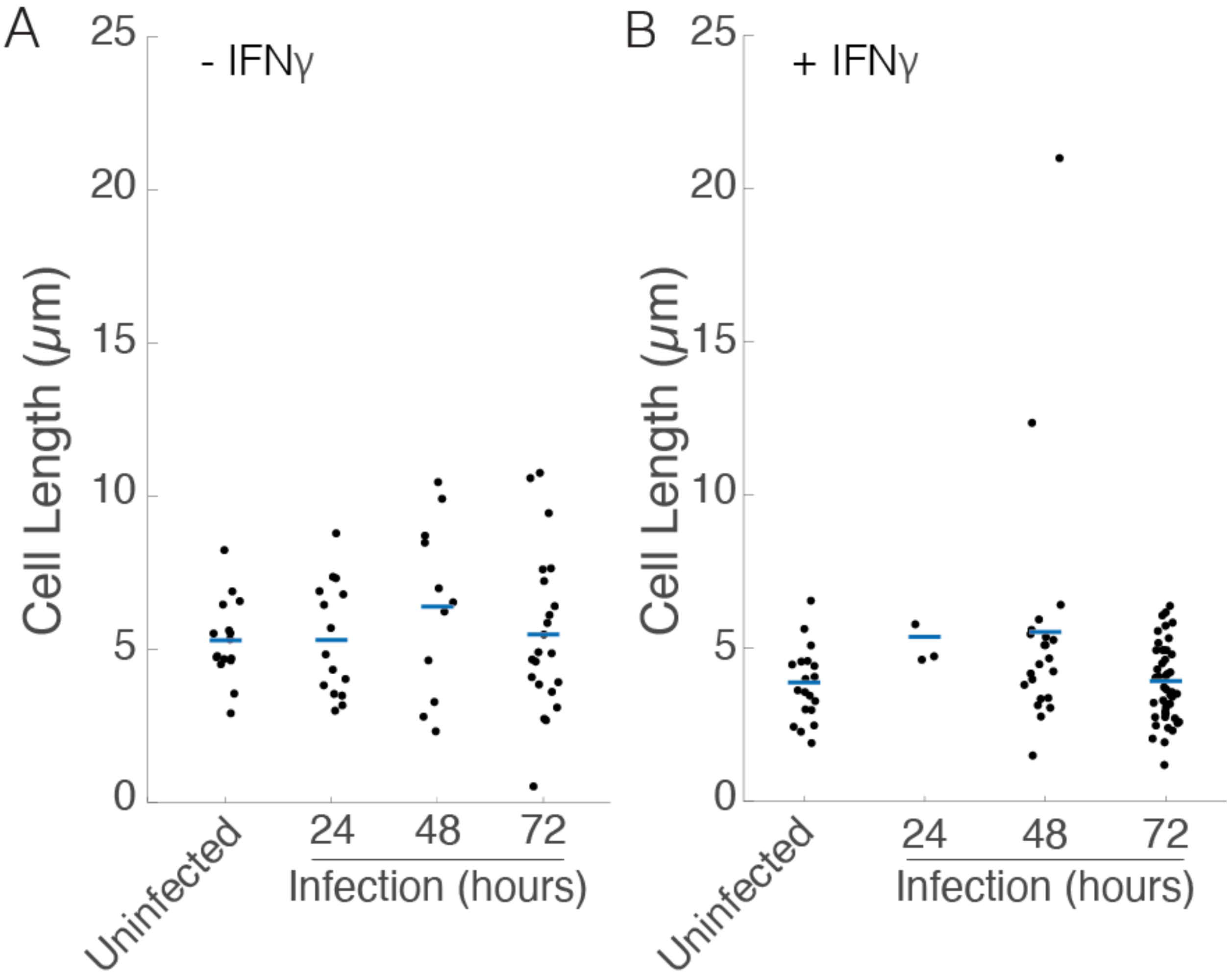

Fig. S6. Mycobacterial cell length heterogeneity.

The length of *M. smegmatis* cells measured by AFM at room temperature (25°C) to probe the mechanical properties in static conditions. Msm bacilli were isolated from macrophages (-/+ IFN-γ) and resuspended in 7H9 growth medium. Measurements are representative of three independent experimental replicates. Blue bars represent the mean of each sample distribution. ~10 – 30 bacteria counted per condition.

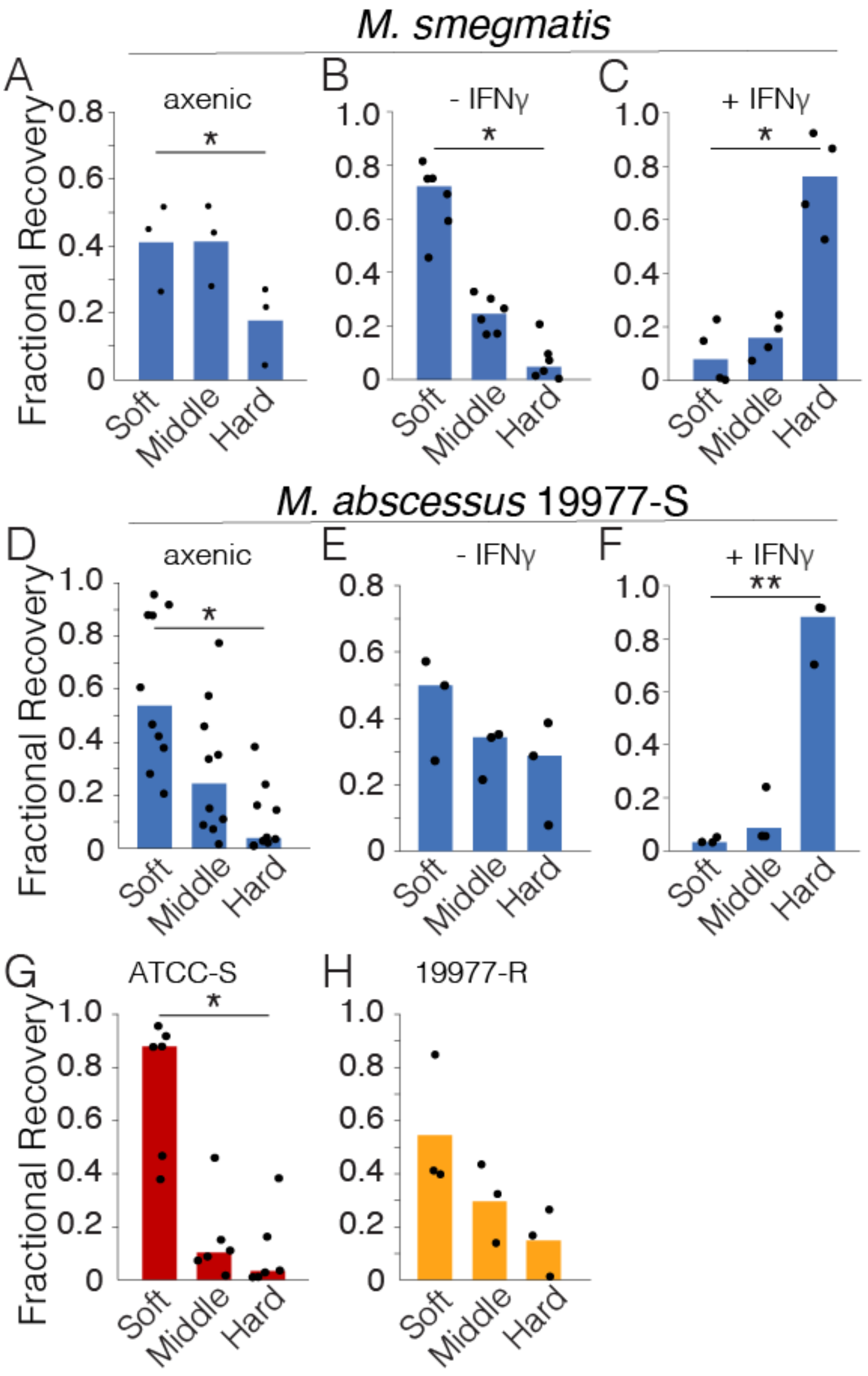

Fig. S7. Distribution of buoyancy of mycobacteria.
M. smegmatis **(A – C)** and *M. abscessus* strain 19977 (smooth morphotype) **(D – F)** fractionated following culturing in 7H9 growth medium (A and D) or isolated from macrophages untreated (B and E) or IFN-γ-treated (C and F). Fractionation of *M. abscessus* strain ATCC (smooth morphotype) (G) and strain 19977 (rough morphotype) (H) grown in 7H9 growth medium. *M. abscessus* 19977-R represents a rough colony morphotype whereas 19977-S and ATCC-S both represent smooth colony morphotype strains. Bars represent mean and dots represent individual experimental replicates. *$P < 0.05$, **$P < 0.01$ by one-way analysis of variance.

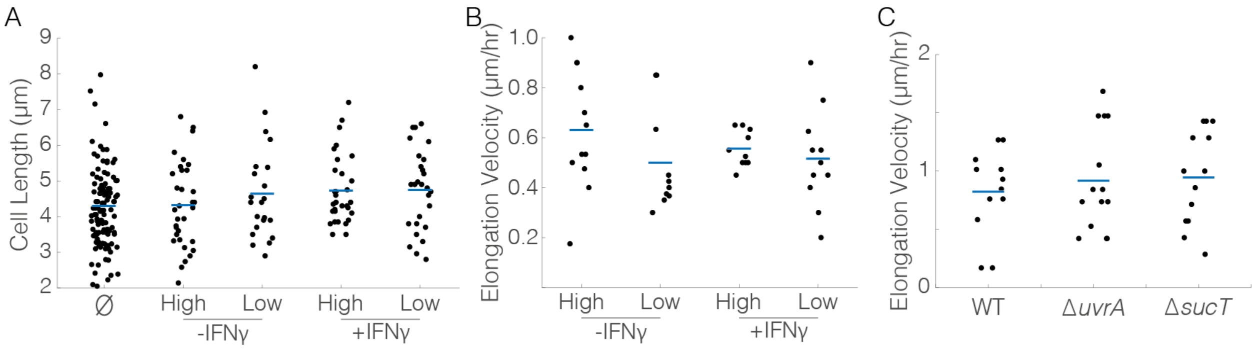

Fig. S8. Cell length and elongation dynamics for Msm bacilli.

Cell length (A) and elongation velocity (B) were measured by LTTL-AFM, of Msm bacilli isolated from BMDMs and fractionated by buoyancy centrifugation. "∅" denotes uninfected Msm. (C) The elongation velocities of Msm mechanical morphotype mutant bacilli exhibit no significant differences from WT Msm. Measurements are representative of three independent experimental replicates. (A) ~30 – 100 bacteria counted per condition. (B & C) ~10 bacteria counted per conditions.

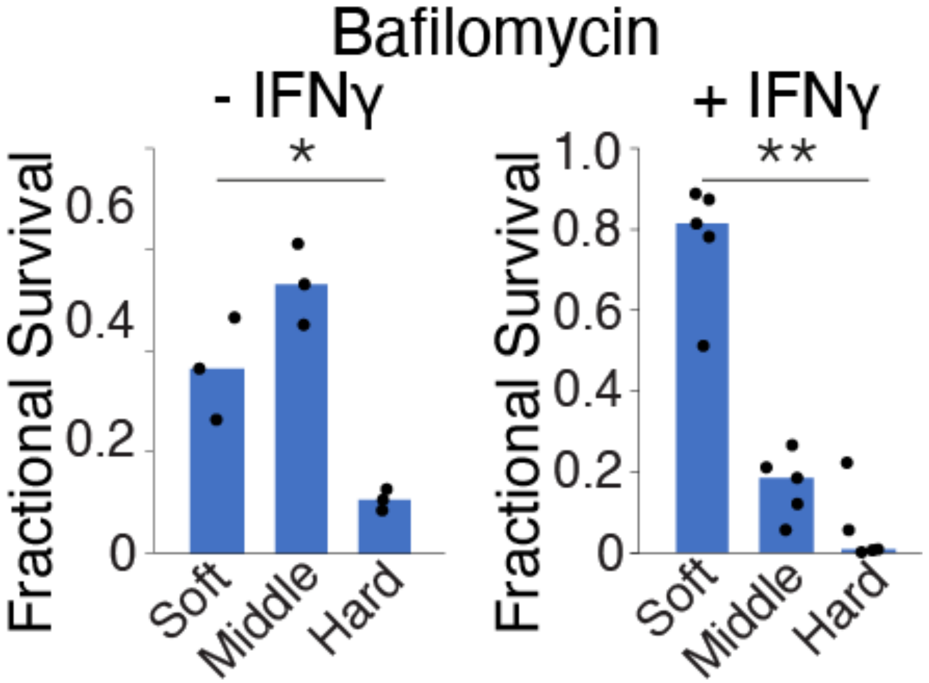

Fig. S9. Vacuolar acidification is necessary for the selection of "high" buoyancy *M. smegmatis* in IFN-γ-stimulated macrophages. Fractional recovery of *M. smegmatis* following buoyancy centrifugation after bacilli were isolated from -/+ IFN-γ-treated macrophages equally treated with Bafilomycin A (10 µM, added at infection start). Bars represent mean and dots represent individual experimental replicates. *$P < 0.05$, **$P < 0.01$ by one-way analysis of variance.

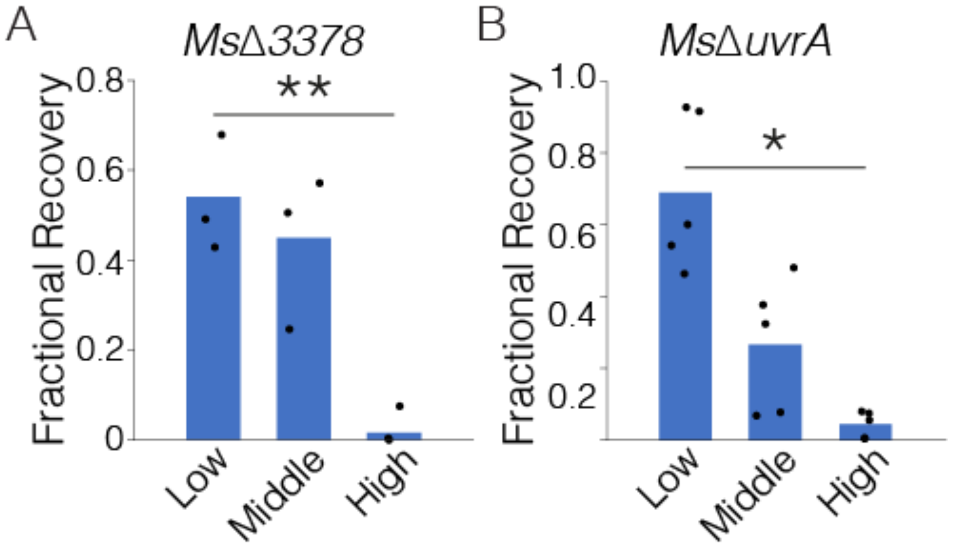

Fig. S10. Fractional recovery of buoyancy fractionated “soft” mechanical morphotype mutants cultured in axenic conditions of growth. Bars represent mean and dots represent individual experimental replicates. *$P < 0.05$, **$P < 0.01$ by one-way analysis of variance.

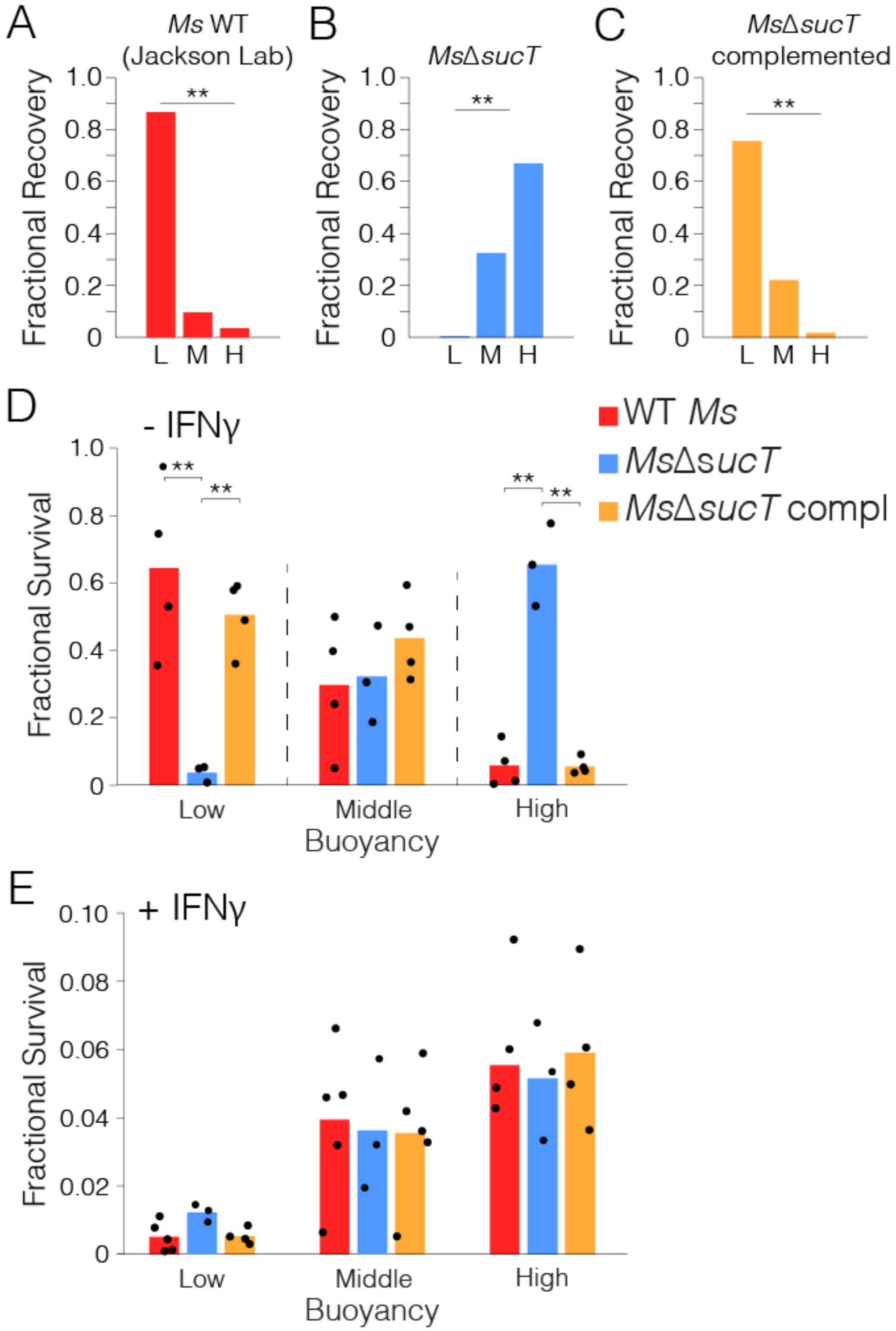

Fig. S11. Relative fractional recovery of *M. smegmatis* Δ*sucT*.

(A – C), The fractional recovery of *M. smegmatis* wildtype (isolate from Colorado State University, "Jackson lab"), Δ*sucT*, and complemented *sucT* cultured in axenic conditions of growth. **(D and E)** Relative fractional recovery of mycobacterial strains isolated from macrophages and buoyancy fractionated. Relative fractional recovery is normalized to input (recovery from macrophages). Bars represent mean and dots represent individual experimental replicates. *$P < 0.05$, **$P < 0.01$ by one-way analysis of variance.

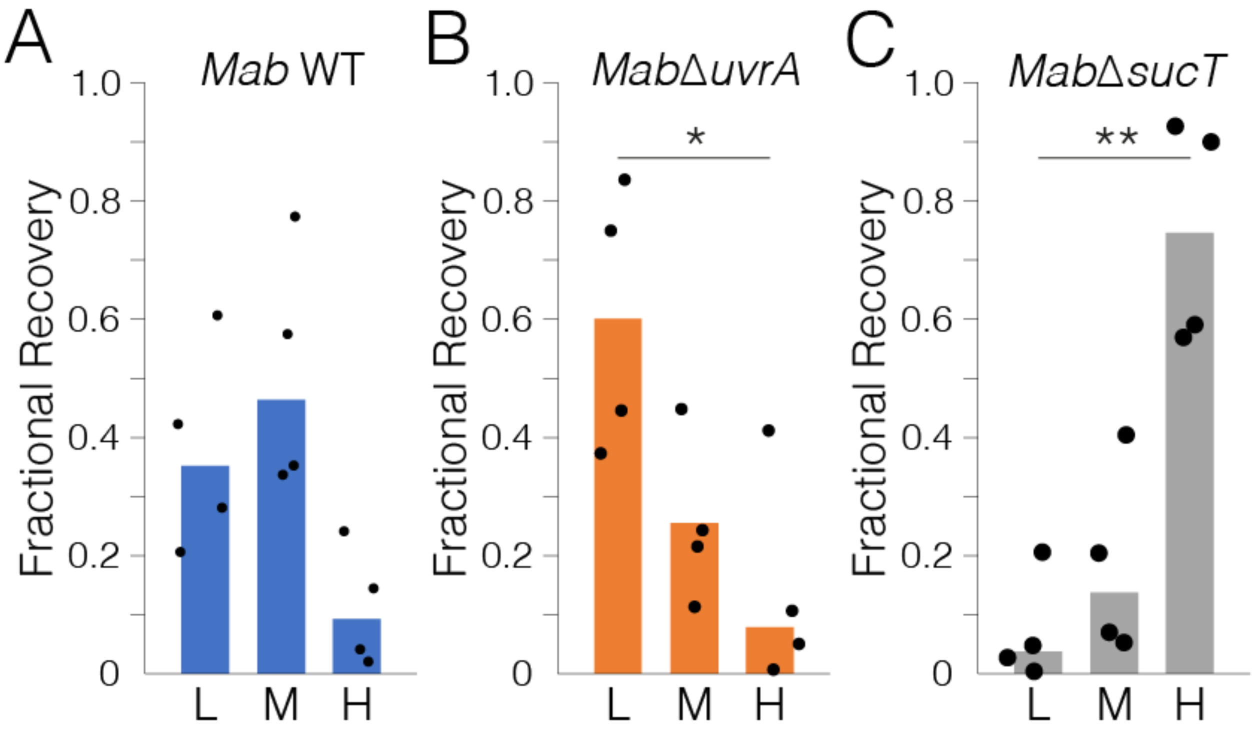

Fig. S12. Fractional recovery of *M. abscessus* mechanical morphotype mutant candidates buoyancy fractionated following culturing in axenic conditions of growth. Bars represent mean and dots represent individual experimental replicates. *$P < 0.05$, **$P < 0.01$ by one-way analysis of variance.

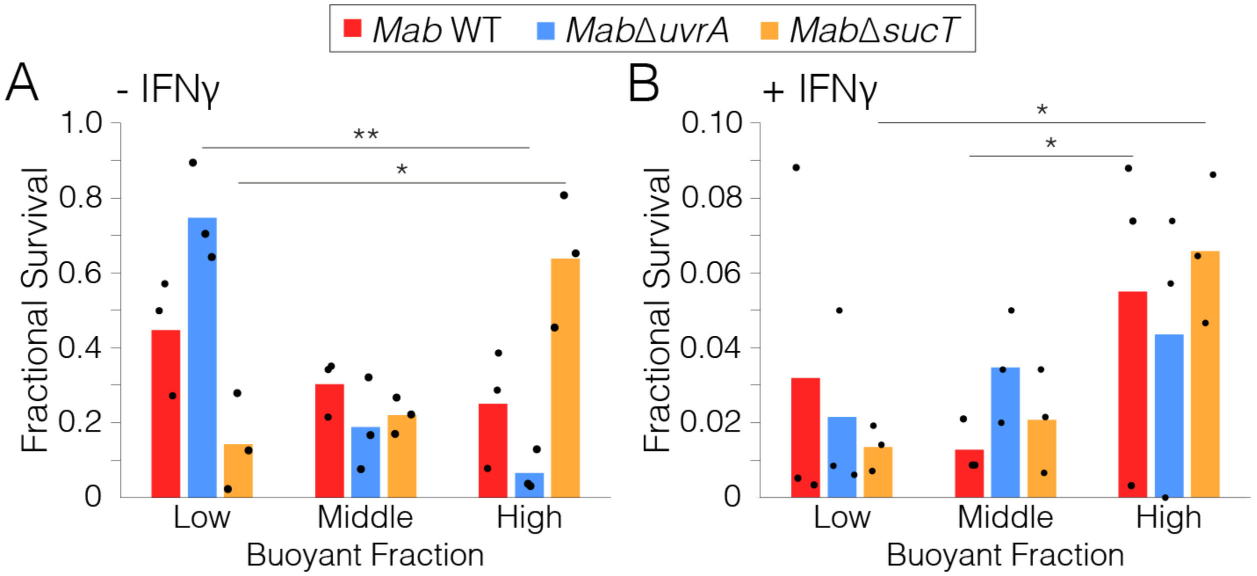

Fig. S13. Distribution in buoyancy for "soft" and "hard" mechano-morphotype mutant candidates following infection of macrophages. Bars represent mean and dots represent individual experimental replicates. *$P < 0.05$, **$P < 0.01$ by one-way analysis of variance.

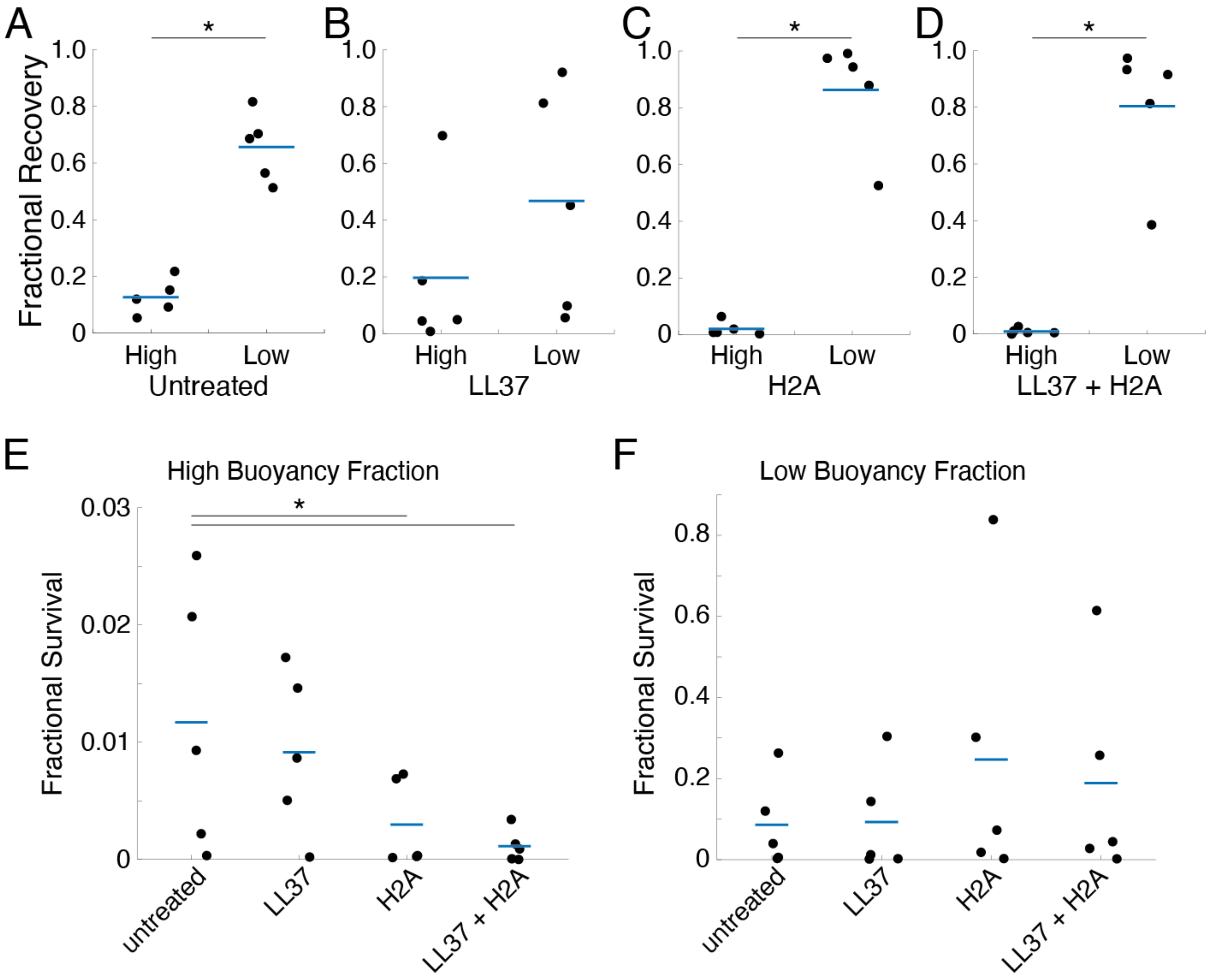

Fig. S14. Treatments of Mab with Cathelicidin and Histone H2A. Wildtype Mab fractionated by buoyancy centrifugation following treatments for 2 hours with LL37 (1µg/ml), H2A (500µg/ml), and in combination. (**A-D**) The distribution of high versus low buoyancy fractions is depicted relative to the sum of the number of bacteria recovered from all buoyancy fractions. (E & F) Relative fractional survival versus input is depicted for high buoyancy fractions (E) and low buoyancy fractions (F), respectively. Bars represent mean and dots represent individual experimental replicates. *$P < 0.05$, **$P < 0.01$ by unpaired T-test (A – D) or one-way analysis of variance test (E).

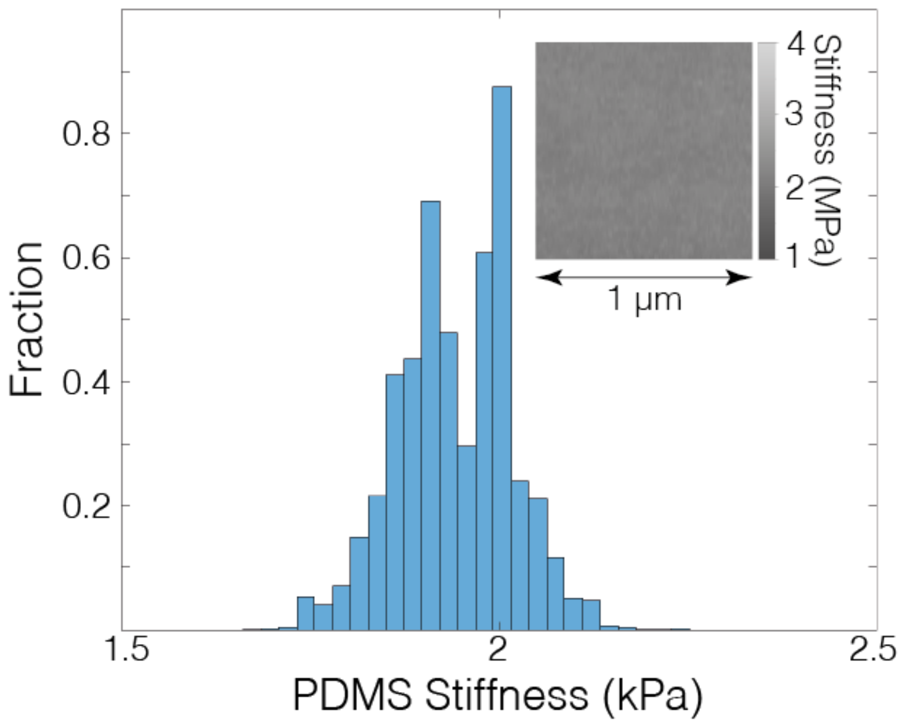

Fig. S15. Calibrating AFM stiffness measurements from DMT modulus images was conducted using the polydimethylsiloxane-coated coverslip sample surface with known Young's modulus of ~1.8 MPa. Histogram depicts the distribution of measurements. Inset AFM DMT modulus image represents the measurements made using the AFM imaging mode: peak force quantitative nanomechanical mapping.

Movie S1. LTTL-AFM imaging of *M. smegmatis* cultured in axenic conditions of growth. AFM imaging was conducted using peak-force off-resonance tapping in time lapse. 3D-Height images are overlayed with the DMT Modulus. *M. smegmatis* was grown at 37°C and the frequency of images taken every 9 minutes. Images represent 15 µm by 7.5 µm of space. The scale of DMT modulus spans 0 – 3 MPa. See Figure 1b and Supplementary Figure 16 for representative schematic images of the same time-lapse.

Movie S2. LTTL-AFM imaging of *M. smegmatis ΔldtAEBCG+F* cultured in axenic conditions of growth. AFM DMT modulus images depicting the cell surface stiffness of a mycobacterial mutant in which all L, D Transpeptidases are absent resulting in less peptidoglycan crosslinking at the new pole defective. The mechanical consequence is bulging spatially localized near the new pole, which happens at a rate that is controlled by the addition of new material at the cell wall (*1*).

Movie S3. LTTL-AFM imaging of *M. smegmatis* WT cells cultured in axenic conditions of growth and antibiotic stress. AFM peak force error images depict the cell surface of *M. smegmatis* cells.

Movie S4. LTTL-AFM imaging of *M. smegmatis* WT cells cultured in axenic conditions of growth and antibiotic stress as per Supplementary Video 3. AFM DMT Modulus images depict the cell surface of *M. smegmatis* cells in conditions of growth, treatment with the bacteriostatic CCCP (5 µM), and recovery of bacilli following washout of the drug.
